# HALNet: Partial Point Cloud Registration Based on Hybrid Attention and Deep Local Features

**DOI:** 10.3390/s24092768

**Published:** 2024-04-26

**Authors:** Deling Wang, Huadan Hao, Jinsong Zhang

**Affiliations:** School of Mechatronic Engineering and Automation, Shanghai University, Shanghai 200444, China; wdl@shu.edu.cn (D.W.); huadanhao@shu.edu.cn (H.H.)

**Keywords:** deep learning, registration, partial point cloud, attention, feature extraction

## Abstract

Point cloud registration is an important task in computer vision and robotics which is widely used in 3D reconstruction, target recognition, and other fields. At present, many registration methods based on deep learning have better registration accuracy in complete point cloud registration, but partial registration accuracy is poor. Therefore, a partial point cloud registration network, HALNet, is proposed. Firstly, a feature extraction network consisting mainly of adaptive graph convolution (AGConv), two-dimensional convolution, and convolution block attention (CBAM) is used to learn the features of the initial point cloud. Then the overlapping estimation is used to remove the non-overlapping points of the two point clouds, and the hybrid attention mechanism composed of self-attention and cross-attention is used to fuse the geometric information of the two point clouds. Finally, the rigid transformation is obtained by using the fully connected layer. Five methods with excellent registration performance were selected for comparison. Compared with SCANet, which has the best registration performance among the five methods, the RMSE(R) and MAE(R) of HALNet are reduced by 10.67% and 12.05%. In addition, the results of the ablation experiment verify that the hybrid attention mechanism and fully connected layer are conducive to improving registration performance.

## 1. Introduction

Abundant geometry information is contained within 3D point clouds, primarily collected by light detection and ganging (LiDAR). Scanning methods of LiDAR are classified as terrestrial or airborne based on its spatial position and further categorized as static or mobile based on its motion status. The typologies scanned by LiDAR mainly consist of urban, rural, and inside buildings. Point cloud data obtained from LiDAR exhibit challenges such as large data volume and variation in point density, necessitating high computational capacity and processing time for data processing algorithms. Machine learning can effectively address these issues. In recent years of research, the applications of machine learning on LiDAR data have encompassed tasks such as building detection, scene segmentation, vegetation detection, and road marking classification [1,2].

LiDAR cannot scan the complete scene information during the data collection process. For instance, when scanning urban ground structures, the scanning range limitations prevent the comprehensive coverage of all ground information in a single scan. Moreover, multiple scans suffer from inconsistent spatial coordinate systems, which makes it difficult to splice multiple scanned images. To acquire a complete scene, it is necessary to align multiple scan images into a unified spatial coordinate system and then obtain a complete scene. Therefore, point cloud registration is a basic task of point cloud data processing and the basis of 3D reconstruction [3,4] and 3D localization [5,6,7].

**Figure 1:**
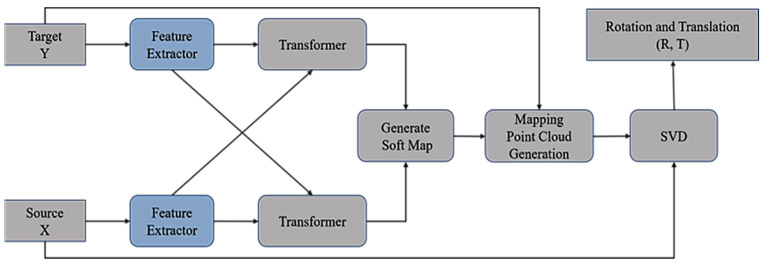
The original framework of DCP. (Y is the target point cloud. X is the source point cloud. R and T are the rotation matrix and translation vector that align the source point cloud with the target point cloud. SVD is an abbreviation for singular value decomposition).

In the process of point cloud registration, point cloud data will have problems such as noise and partial point cloud loss, which have a great impact on the registration accuracy. Many learning-based registration methods [8,9,10,11] have shown good registration performance in the complete point cloud scene, but their registration performance is poor when dealing with the task of partial point cloud registration. HALNet, a learning-based end-to-end partial point cloud registration network based on hybrid attention and deep local features, is proposed in this paper with reference to the registration model proposed in DCP [8] (as shown in Figure 1).

HALNet consists of four modules: feature extraction module, overlap prediction, feature rectification, and transformation prediction module. The feature extraction module consists of two parallel networks, one learning local features and the other extracting global features. Global features represent the overall structure and distribution information of the entire point cloud, such as its overall shape, size, and orientation. These features facilitate rapid matching and localization of the entire point cloud, thus providing initial references for registration. Local features within the point cloud concentrate on detailed information like local shapes and edges, enabling differentiation between different parts and facilitating the identification and matching of regions with similar structures, leading to more precise registration. The AGConv [12] is used to extract the local features of the point cloud and embed them into the high dimension feature space, and the CBAM [13] is used to enhance the local features. The global features of the point cloud are obtained by a convolution layer composed of multiple two-dimensional convolutions. The point cloud feature extracted by the feature extraction module is used by the overlap prediction module to calculate the overlap point set between the source point cloud and the target point cloud. The hybrid attention mechanism is used to learn the interrelated features within the point cloud and the structural features between the source point cloud (target point cloud) and the target point cloud (source point cloud). The overlapping point set features processed by the hybrid attention mechanism will be used as the residual term to correct the overlapping point set features. The rigid transformation-solving module is composed of a fully connected layer. The feature grouping function of the full connection layer enables the registration network to obtain a rotation matrix and translation vector that align the source point cloud and the target point cloud. The main contributions of this paper are as follows:

1. An end-to-end partial point cloud registration network, HALNet, is proposed. The network can extract deep local features through AGConv and CBAM and use the similarity scores of the point pairs of source and target point clouds to remove non-overlapping points in two point clouds.
2. A hybrid attention mechanism composed of self-attention and cross-attention is proposed to modify the extracted features and help to improve the accuracy of feature grouping for predicting rigid transformation.

## 2. Related Work

Research on point cloud registration based on deep learning mainly falls into two categories: feature-learning-based point cloud registration and end-to-end point cloud registration. The main idea of the feature-learning-based method is to utilize deep learning networks to learn deep features for estimating accurate correspondences, followed by employing a differentiable singular value decomposition (SVD) or random sample consensus (RANSAC) to estimate rigid transformations. The end-to-end method addresses registration problems by neural network only. This method takes two point clouds as input and outputs the rotation matrix and translation vector aligning these two point clouds. In other words, the transformation estimation is performed by the neural network, distinguishing it from the feature-learning method where feature learning and transformation estimation are separated. Various attention mechanisms are employed in feature learning or matching point pairs for point cloud registration to enhance registration performance. These attention mechanisms were adapted from image processing and specifically designed for point cloud features.

### 2.1. Feature-Learning-Based Methods

AlexNet is utilized by 3DMatch [14] to learn 3D features from RGB-D datasets. In the workflow of 3DMatch, the first step involves converting 3D point cloud data into 3D voxel data, which is then fed into a neural network to extract local features. These extracted local features contain characteristics of representative points in the local region and their surrounding structures. 3DSmoothNet [15] introduces a novel compact learning-based local feature descriptor for point cloud matching. This method uses smooth density value (SDV) voxelization to generate an SDV voxel grid as the input representation for the feature learning network, saving network capacity by learning highly descriptive features. Unlike providing volumetric data to the feature learning network, PPFNet [16] learns only geometric local descriptors and has a strong understanding of global context. 3DFeatNet [17] introduces a weakly supervised method for learning 3D feature detectors and point cloud descriptors. This method utilizes alignment operations and attention mechanisms to learn feature point matching for 3D point clouds labeled with a Global Positioning System/Inertial Navigation System (GPS/INS). RPMNet [18] introduces a more robust deep-learning-based point cloud registration method that is less sensitive to initialization. The network of this method can obtain soft assignments of point correspondences, addressing the issue of local visibility in point clouds. DCP [8] employs dynamic graph convolutional neural networks for feature extraction and uses attention modules to generate new embeddings that consider the relationship between two point clouds. IDAM [9] combines geometric and distance features into the iterative matching process to learn features of partially overlapping point clouds.

### 2.2. End-to-End Methods

Deng et al. [19] proposed RelativeNet to directly estimate poses from features. Lu et al. [20] proposed DeepVCP, which automatically avoids dynamic objects and selects easily matchable key points by incorporating semantic features. Within the candidate matching region of key points, same-name points are generated by calculating the probability of feature similarity, and the network loss comprehensively integrates the local and global matching effects of key points. PointNetLK [21] employs PointNet [22] to extract global features of two input point clouds, then utilizes the Iterative Closest Point (ICP) algorithm to estimate the transformation matrix. The objective is to minimize the feature discrepancy between the two features by estimating the transformation matrix. DeepGMR [10] uses neural networks to learn the correspondence between pose-invariant points and distribution parameters. These correspondences are then fed into a GMM optimization module to estimate the transformation matrix. Elbaz et al. [23] proposed an auto-encoder-based registration network that combines super-points extraction and unsupervised feature learning. FMR [24] introduced a fast feature-metric point cloud registration framework, which performs registration optimization by minimizing the projection error of feature metrics without corresponding relationships.

### 2.3. Attention Mechanism

The application of the attention mechanism is already a common way to solve problems in visual tasks, and the learning of point cloud features also introduces the attention mechanism. Yang et al. [25] proposed Pointment Attention Transformers (PATs), using parametric-efficient group shuffling attention instead of a multi-head attention mechanism to learn the relationship between points. Zhang et al. [26] proposed a Point Contextual Attention Network (PCAN), based on the point context, which can predict the importance of local point features and pay more attention to task-related features when aggregating local features. The GAPNet proposed by Chen et al. [27] learns the attention features of each point by highlighting different attention weights in the neighborhood and uses a multi-head mechanism to aggregate different features from independent heads. Zhao et al. [28] designed a self-attention layer for the point cloud and used it to build a self-attention network for different task scenarios. Lu et al. [29] designed the spatial-channel attention module, which selects differentiated features according to the generated spatial and channel attention, effectively combining multi-scale and global information. Guo et al. [30] proposed the PCT network, using offset attention instead of the existing self-attention as the attention module of the PCT network so that it has better semantic feature learning ability.

## 3. Methodology

**Figure 2:**
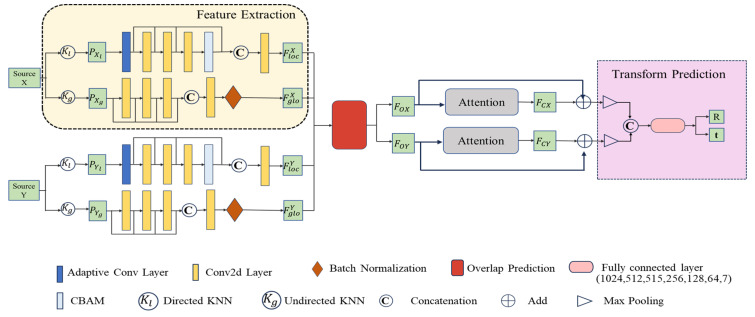
Network architecture of HALNet. (The input of HALNet is the source and target point clouds to be registered, and the output is the rotation matrix **R** and translation vector **t** that align the source point cloud X to the coordinate system of target point cloud Y. $P_{X_{l}}$ and $P_{X_{g}}$ represent the global and local structures used to extract the global and local features of X, respectively. And $P_{Y_{l}}$ and $P_{Y_{g}}$ are the same for Y. $F_{glo}^{X}$, $F_{clo}^{X}$, $F_{glo}^{Y}$, and $F_{clo}^{X}$ represent global and local features corresponding to X and Y, respectively. $F_{OX}$ and $F_{OY}$ are the features of X and Y without non-overlapping points. $F_{CX}$ and $F_{CY}$ are features that include local details and incorporate geometry of X and Y).

The registration network’s capability to learn point cloud features has a significant impact on registration performance, particularly for partial point cloud registration. Learning rich features and selecting overlapping point sets are critical for HALNet to demonstrate excellent registration performance. Additionally, leveraging attention to assign higher weights to important features and mitigate interference from irrelevant features, along with exploiting the feature classification ability of fully connected layers, has enhanced registration accuracy. The network architecture of HALNet is shown in Figure 2. Note that all of the following equations refer to expressions commonly used in similar studies [8,12,31,32,33].

### 3.1. Preliminary

The purpose of point cloud registration is to estimate the optimal rigid transformation that aligns two given point clouds, $X=\left\{x_{i}|i=1,2,...,N_{x}\right\}\in R^{N_{x}\times 3}$ and $Y=\left\{y_{i}|i=1,2,\ldots ,N_{y}\right\}\in R^{N_{y}\times 3}$. Here, $x_{i}$ and $y_{i}$ represent the $\left(x,\;y,\;z\right)$ coordinates of the *i*-th point in X and Y, respectively. $N_{x}$ and $N_{y}$ are the number of points in X and Y, and for convenience of description, assume that $N_{x}$ is equal to $N_{y}$. In the point cloud registration process, X is defined as the source point cloud, and Y is defined as the target point cloud. Assuming that Y results from applying a rigid transformation to X, this transformation can be represented by a rotation matrix R⊂SO(3) and a translation vector $t\subset R^{3}$. The rotation matrix R is obtained from quaternion q=w+xi+yj+zk through Equation (1).

(1) $$R=\left[\begin{array}{ccc} 1-2y^{2}-2z^{2} & 2xy-2wz & 2xz+2wy\\ 2xy+2wz & 1-2x^{2}-2z^{2} & 2yz-2wx\\ 2xz+2wy & 2yz+2wx & 1-2x^{2}-2y^{2} \end{array}\right]$$

The translation vector t is obtained from Equation (2).

(2) $$t=\left[\begin{array}{c} t_{x}\\ t_{y}\\ t_{z} \end{array}\right]$$

where $t_{x}$, $t_{y}$, $t_{z}$ are the three translation parameters.

### 3.2. Feature Extraction

The structure of the feature extraction network is shown in Figure 2, consisting of two parallel networks for extracting local and global features. The input point cloud data record the 3D coordinates of each point, where multiple points collectively form the overall shape of the point cloud. Since the given point cloud data only contain the 3D coordinates of each point, the k-nearest neighbors (KNN) algorithm is employed to construct the graph of the point cloud, denoted as $K_{l}\;$and $K_{g}$ in Figure 2. $K_{l}$ is constructed as a directed graph for extracting local features, while $K_{g}$ is constructed as an undirected graph for extracting global features.

We define the input point cloud as $P=\left\{p_{i}|i=1,2,\ldots ,N\right\}\in R^{Nx3}$, where $p_{i}$ represents the $\left(x,\;y,\;z\right)$ position of the *i*-th point, and its corresponding feature is $F=\left\{f_{i}|i=1,2,\ldots ,N\right\}\in R^{N\times D}$, where D is the feature dimension of the *i*-th point. Based on the provided point cloud, one graph G=(ν,ξ) is computed, where ν={1,…,N} and ξ⊆(ν×ν) represent the vertices and edges, and the set of point indexes in the domain of the local area is represented as $S\left(i\right)=\left\{j:\left(i,j\right)\in \xi \right\}$.

The local feature extraction network mainly consists of AGConv [12] and CBAM [13]. The input point cloud data, after computation by $K_{l}$, yield $∆p_{ij}$, which is first embedded into a high-dimensional feature space by the AGConv. Compared to conventional convolution, AGConv generates adaptive convolutional kernels based on dynamically learned point features. The uniqueness of the relationships between the central point and each neighboring point ensures that the learned point features in the local region possess uniqueness. The generation process of adaptive convolutional kernels can be represented as:

(3) $$Kernels=conv2d\left(∆p_{ij}\right)$$

where $∆p_{ij}=\left[p_{i},p_{j}-p_{i}\right]$, $p_{i}$ represents the spatial position information of a local region composed of *K* points, $p_{j}$ represents the spatial position information of neighboring points, $\left[\cdot ,\cdot \right]$ is concatenation operation, and conv2d is a mapping function that embeds low-dimensional features into high-dimensional feature spaces.

The features embedded in the high-dimensional space undergo three layers of 2D convolution operations and then are fed into CBAM for feature enhancement. Skip connections are introduced in this network to concatenate the output of each layer, ensuring that the details of the point cloud features are preserved during the learning process. After concatenation, the features undergo another layer of 2D convolution operations to obtain the local features of the point cloud $F_{loc}=\left\{f_{loci}|i=1,2,\ldots ,N\right\}\in R^{N\times M}$. The specific process described above can be represented as:

(4) $$f_{loci}=\sigma \left(conv2d\left[f_{i}^{M_{0}},pool(f_{i}^{M_{1}}),pool(f_{i}^{M_{2}}),pool(f_{i}^{M_{3}})\right]\right)$$

where $f_{loci}$ is local feature of the point, σ is activation function, pool is pooling operation that reduces the number of parameters to prevent overfitting, $f_{i}^{M_{1}}=conv2d\left(f_{i}^{M_{0}}\right)$, $f_{i}^{M_{2}}=conv2d(f_{i}^{M_{1}})$, $f_{i}^{M_{3}}=Att(conv2d\left(f_{i}^{M_{2}}\right))$, $f_{i}^{M_{0}}=\sigma \left\langle Kernels,\right.\left.∆p_{ij}\right\rangle$, $\langle \cdot ,\cdot \rangle$ is inner product of output vectors, Att is CBAM, and $M_{0},\;M_{1},\;M_{2},M_{3}\;$represent the number of feature channels for each layer of convolutional output, respectively.

The global feature extraction network mainly consists of four layers of 2D convolution. The input point cloud data, after computation by $K_{g}$, yield $p_{ij}$, which first undergoes three layers of 2D convolution operations. Similar to the learning network for local features, this network also introduces skip connections. The concatenated features are then subjected to one layer of 2D convolution operation and normalization to obtain the global features of the point cloud $F_{glo}=\left\{f_{gloi}|i=1,2,\ldots ,N\right\}\in R^{N\times C}$. The specific process described above can be represented as:

(5) $$f_{gloi}=\sigma \left\{BN\left(conv2d\left[p_{ij},pool\left(f_{i}^{C_{1}}\right),pool\left(f_{i}^{C_{2}}\right),pool\left(f_{i}^{C_{3}}\right)\right]\right)\right\}$$

where $f_{gloi}$ is global feature of the point, $f_{i}^{C_{1}}=conv2d\left(p_{ij}\right)$, $p_{ij}=\left[p_{i},\;p_{j}\right]$, $f_{i}^{C_{2}}=conv2d(f_{i}^{C_{1}})$, $f_{i}^{C_{3}}=conv2d\left(f_{i}^{C_{2}}\right)$, BN is batch normalization, and $C_{1},\;C_{2},C_{3}$ represent the number of feature channels for each layer of convolutional output, respectively.

### 3.3. Overlapping Region Estimation

In partial point cloud registration, the features of the overlapping regions have the greatest impact on registration accuracy. To achieve higher registration accuracy, it is necessary to estimate the overlapping regions of the source and target point clouds. The overlap area is calculated by using the estimated overlap score to determine whether a point needs to be removed, i.e., whether a point belongs to an overlap area based on the overlapping score. The operation of removing overlapping regions can also be referred to as pruning and is a bidirectional operation performed simultaneously on both the source and target point clouds, rather than being performed on a single point cloud.

The feature extraction module outputs local and global features corresponding to the source and target point clouds. Local features $F_{loc}^{X_{i}}\in F_{loc}^{X}$ and $F_{loc}^{Y_{j}}\in F_{loc}^{Y}$ of each point are concatenated with global features $F_{glo}^{X}$ and $F_{glo}^{Y}$, respectively, and then fed into the shared multi-layer perceptron (MLP) for estimating overlap consistency scores:

(6) $$S^{X_{i}}=g_{\emptyset }\left(\left[F_{loc}^{X_{i}},\delta \left(F_{glo}^{X}\right),\delta \left(F_{glo}^{Y}\right)\right]\right)$$

(7) $$S^{Y_{j}}=g_{\emptyset }\left(\left[F_{loc}^{Y_{j}},\delta \left(F_{glo}^{X}\right),\delta \left(F_{glo}^{Y}\right)\right]\right)$$

where $S^{X_{i}}$ is the score of the coincidence probability between i-th point in X and i-th in Y, $S^{Y_{j}}\;$is the score of the coincidence probability between j-th point in Y and j-th in X, $g_{\emptyset }\left(\cdot \right)$ is shared MLP, $\delta \left(\cdot \right)$ is the channel-wise repeat operation, and $\left[\cdot ,\cdot \right]$ is concatenation operation.

Based on the obtained overlap consistency scores, the first N points are retained and corresponding features are obtained. The retained points and corresponding features are denoted as $X_{O},Y_{O}\in R^{\hat{N}\times 3}$ and $F_{OX},F_{OY}\in R^{\hat{N}\times d}$, respectively.

(8) $$X_{O}=X\left[\underset{\mathrm{top}-\hat{N}}{\mathrm{Indices}}\left(S^{X}\right)\right],F_{OX}=F_{loc}^{X}\left[\underset{\mathrm{top}-\hat{N}}{\mathrm{Indices}}\left(S^{X}\right)\right]$$

(9) $$Y_{O}=Y\left[\underset{\mathrm{top}-\hat{N}}{\mathrm{Indices}}\left(S^{Y}\right)\right],F_{OY}=F_{loc}^{Y}\left[\underset{\mathrm{top}-\hat{N}}{\mathrm{Indices}}\left(S^{Y}\right)\right]$$

where $\underset{\mathrm{top}-\hat{N}}{\mathrm{Indices}}\left(\cdot \right)\;$represents to obtain top $\hat{N}$ indices based on $S^{X}$ or $S^{Y}$.

### 3.4. Hybrid Attention

**Figure 3:**
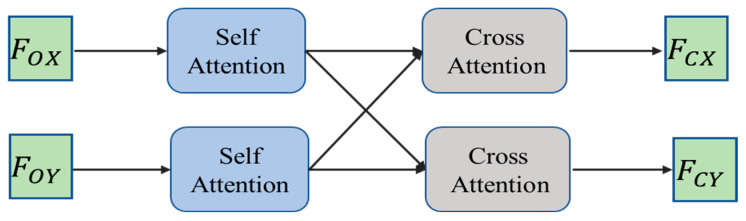
The structure of hybrid attention. The inputs $F_{OX}$ and $F_{OY}$ are the features of the overlapping point set, and the outputs $F_{CX}$ and $F_{CY}$ are the features containing the internal features and the geometric structure of the two point clouds.

The self-attention and cross-attention are combined to form a hybrid attention mechanism, which enables the registration network to learn the features of the point cloud context. The structure of the hybrid attention is shown in Figure 3.

#### 3.4.1. Self-Attention

For machine learning tasks, the ability of a model to capture discriminative features from a plethora of given information is crucial for the target task. As a variant of attention mechanisms, self-attention enables models to focus on important aspects, making them widely applicable in machine learning tasks such as machine translation, speech recognition [34], and caption generation [35]. The principle of self-attention can be represented as:

(10) $$Attention(Q,K,V)=Softmax\left(\frac{QK^{T}}{\sqrt{d_{k}}}\right)V$$

where Q is query, K is key, V is value, and $d_{k}$ is the dimension of the parameter V.

The introduction of self-attention enables the point cloud features used for estimating rigid transformations to incorporate internal contextual information. At the same time, it helps filter out features that are difficult to match from a multitude of features and weaken their influence. Using the overlapping features $F_{OX}\in R^{\hat{N}\times d}$ of the source point cloud X as an example, the specific implementation of self-attention in the registration network is described. Firstly, compute Q**, *K***, and ***V***:

(11) $$Q=W_{Q}F_{OX},\;K=W_{K}F_{OX},\;V=W_{V}F_{OX}$$

where $W_{Q}$, $W_{K}$, and $W_{V}$ are learnable weight matrices. Based on the obtained Q and K, calculate the self-correlation weight matrix of features:

(12) $$W_{Att}=Softmax\left(QK^{T}\right)\in R^{\hat{N}\times \hat{N}}$$

Multiply the obtained self-correlation weight matrix with V to obtain the source point cloud features with incorporated self-correlation information.

(13) $$F_{SX}=W_{Att}V$$

The overlapping region features of the target point cloud will undergo the same calculation process, resulting in the target point cloud features $F_{SY}$ with incorporated self-correlation information.

#### 3.4.2. Cross-Attention

The cross-attention is an extension of the self-attention. Unlike the self-attention where the inputs come from the same sequence, the cross-attention takes inputs from different sequences. Apart from this distinction, the calculation process is similar to the self-attention.

For registration tasks, finding point pairs in the target point cloud that are highly correlated with the source point cloud is one of the crucial steps. While self-attention enables the registration network to capture dependencies within individual point clouds, it fails to capture the correlation between the source and target point clouds. The cross-attention is concatenated with the self-attention to address this issue. This ensures that the final learned features of the source point cloud contain both internal correlation information and learned external correlation information with the target point cloud, and vice versa for the target point cloud.

For cross-attention, ***K*** and ***V*** result from one input, while Q results from another input. Firstly, the calculation of the source point cloud features in the cross-attention is described. The first step is to compute the correlation matrix $W_{CX}$ between the source point cloud and the target point cloud:

(14) $$W_{CX}=Softmax\left(\frac{F_{SY}{F_{SX}}^{T}}{\sqrt{d_{k}}}\right)\in R^{\hat{N}\times \hat{N}}$$

And then, multiply $W_{CX}$ with $F_{SX}$ to obtain the source point cloud features with incorporated information related to the target point cloud:

(15) $$F_{CX}=W_{CX}F_{SX}$$

The processing steps for the target point cloud are similar. The correlation matrix between the target point cloud and the source point cloud is denoted as $W_{CY}$:

(16) $$W_{CY}=Softmax\left(\frac{F_{SX}{F_{SY}}^{T}}{\sqrt{d_{k}}}\right)\in R^{\hat{N}\times \hat{N}}$$

The target point cloud features with incorporated information related to the source point cloud are denoted as $F_{CY}$:

(17) $$F_{CY}=W_{CY}F_{SY}$$

### 3.5. Rigid Transformation Calculation

The fully connected layers are employed to classify the learned point cloud features, ultimately obtaining the rotation matrix and translation vector that align the source point cloud with the target point cloud. The specific process is described as follows.

The features generated by the feature extraction network are filtered by the overlapping estimation module to select the features of the overlapping point set. To ensure that the point cloud features used for registration have more complete structural information, the features obtained through hybrid attention are used as residual terms to correct the overlapping features.

(18) $$\Phi_{X}=F_{OX}+F_{CX}$$

(19) $$\Phi_{Y}=F_{OY}+F_{CY}$$

The corrected features of the source point cloud and target point cloud are concatenated after undergoing max pooling operations. The concatenated features are then fed into fully connected layers for feature classification.

(20) $$Pose=FC\left[pool\left(\Phi_{X}\right),pool\left(\Phi_{Y}\right)\right]$$

where Pose is the output of fully connected layers; FC is fully connected layers. There are a total of six fully connected layers for feature classification, as shown in Figure 2. Pose is a seven-dimensional vector, with the first four dimensions representing the quaternion for rotation and the last three dimensions representing the translation vector. The procedure for converting a quaternion to a rotation matrix is described in Section 3.1.

Compared to using SVD to obtain rigid transformation, the fully connected layers with learnable weights and bias parameters can be optimized through backpropagation. This optimization enables the registration network to better fit the training data, thereby improving the registration accuracy.

### 3.6. Loss Function

The objective is to align the provided point clouds by accurately predicting the transformation. Hence, the predicted transformation and the true transformation are selected as variables to formulate the loss function, which is defined as Equation (21).

(21) $$Loss={∥R_{p}^{T}R_{g}-I∥}^{2}+{∥t_{p}-t_{g}∥}^{2}+\lambda {∥\theta ∥}^{2}$$

where $R_{g}$ and $R_{p}$ denote ground truth rotation matrix and predicted rotation matrix, respectively, and $t_{g}$ and $t_{p}$ denote ground truth translation vector and predicted translation vector. The first two terms define a simple distance on SE (3), and the third term represents the Tikhonov regularization of the registration network parameter *θ*.

## 4. Experiments

The experiments were divided into performance evaluation and ablation study. In the performance experiments, HALNet was compared with five deep-learning-based methods to validate its registration performance in partial point cloud registration. The ablation experiments examined the influence of hybrid attention and fully connected layers on registration performance.

### 4.1. Experimental Setup

All experiments were conducted on a 64-bit Windows operating system, utilizing an NVIDIA GeForce RTX4080 GPU mading by NVIDIA (Santa Clara, CA, USA) and buying from the company’s China sales department. The framework employed for the experiments was PyTorch version 1.12.1. The initial learning rate for network training was set to 1 × 10^−4^. The Adam optimizer [36] was utilized to optimize the network parameters over 300 training epochs.

### 4.2. Dataset

The dataset used in this paper was ModelNet40 [37], which consists of 12,311 meshed CAD models from 40 different categories. Of these, 9843 models were used for training in the experiment, and 2468 models were used for testing. Each source point cloud was randomly rotated within the range of [0°, 45°] and randomly translated within the range of [−0.5, 0.5]. The resulting point cloud after transformation served as the target point cloud corresponding to the source point cloud. Then, for both the source and target point clouds, a nearest neighbors search algorithm was employed to randomly select 1024 points as the source and target point clouds for partial point cloud registration.

### 4.3. Evaluation Indicators

The root mean square error (RMSE) and the mean absolute error (MAE) between the ground truth and the predicted value were used as evaluation indicators for the experimental results. Ideally, if the rigid alignment is perfect, all of the above error measures should be zero. When evaluating the registration performance, lower values of the evaluation metrics indicate better registration performance for the corresponding method.

### 4.4. Performance Evaluation

HALNet was compared with DeepGMR [10], DCP [8], IDAM [9], SCANet [38], and VRNet [11]. The experimental results for each method were obtained by training with the source code provided by the respective authors.

#### 4.4.1. Unseen Shapes

The unseen shapes experiment refers to having the same categories in both the training and testing datasets but using different shapes for training and testing. Additionally, due to secondary processing of the data, the point cloud data do not represent complete shapes, making the specific shapes within the same category unknown. There were 9843 objects in the training set and 2468 objects in the testing set. This experiment validates the partial point cloud registration performance of the registration methods. The final registration experiment results are shown in Table 1, with underlines indicating the lowest values.

From the experimental results presented in the table, it is evident that HALNet outperformed the comparative methods in partial point cloud registration performance. Among all the compared methods, DeepGMR exhibited the poorest performance in partial point cloud registration, with significantly higher rotation evaluation metric values compared to the other methods. Although the RMSE(t) and MAE(t) of HALNet were not the lowest, ranking second after SCANet, considering the overall results of rotation and translation evaluation metrics, HALNet still demonstrated excellent registration performance.

The registration performance of the above comparison method was inferior to that of HALNet for the following two reasons: First, the feature extraction network used was different. For example, DeepGMR used shared MLP to extract the global features of the point cloud, while ignoring the impact of local features on the registration performance. SCANet extracted local information and global information at different levels using a network composed mainly of self-attention mechanism and fully connected layer. However, it did not use the local graph of points to learn local information, resulting in the loss of local details. HALNet first constructed the local relationship graph of points. Then AGConv and CBAM were used to extract rich local features. The second point is the selection of overlapping points. After learning the features of point cloud, HALNet removed non-overlapping points to reduce the interference to registration, but other comparison algorithms ignored the impact of non-overlapping points on registration performance.

**Figure 4:**
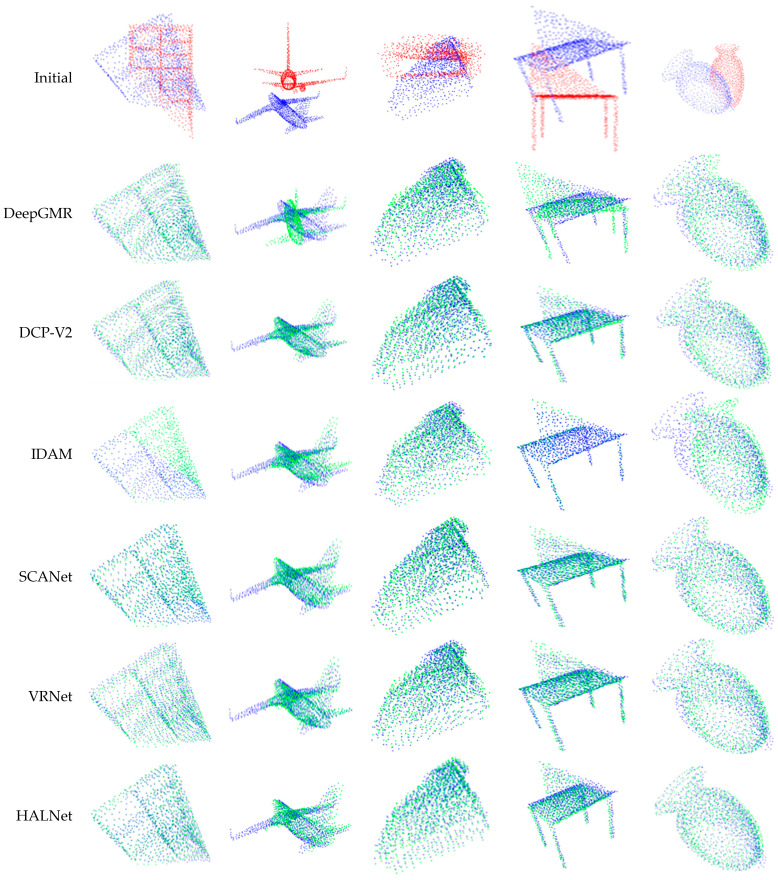
Registration results of unseen shapes (red: source point cloud; blue: target point cloud; green: predicted point cloud). The first row shows the initial position of the source and target point clouds, and the other rows show the registration results.

In order to give readers a more intuitive understanding of the registration performance, five point clouds in the testing datasets were randomly selected to obtain the registration results obtained by different methods. The result is shown in Figure 4.

#### 4.4.2. Noise

Point clouds collected from sensing devices may contain noise and outliers due to measurement errors. To verify the robustness, the noise was sampled from $N\left(0,\;0.01\right)$, clipped to the range $\left[-0.05,\;0.05\right]$, and then added to point cloud X and Y for testing. The dataset used was the same as that in the unseen shapes experiment. The model used in this experiment was obtained from the unseen shapes. The experimental results after adding noise are shown in Table 2.

Comparing the experimental results from Table 1 and Table 2, it was observed that, except for DeepGMR which showed minimal change in registration performance, the registration performance of other methods generally declined after adding noise. However, HALNet still outperformed the other comparative methods in terms of registration accuracy. Despite a slight decrease in registration performance after the addition of noise, HALNet remained robust to noise.

#### 4.4.3. Unseen Categories

Unseen categories refers to the situation where the object categories seen in the training set do not appear in the testing set. Since the training dataset cannot contain all data categories, evaluating the generalization of the registration network was necessary. In this experiment, the first 20 categories from ModelNet40 were used for the training set, and the remaining 20 categories were used for the testing set. The results of the point cloud registration experiment with unseen categories are shown in Table 3.

The results listed in Table 3 indicate that, compared to the unseen shapes registration results, the registration performance of each method decreased. DeepGMR almost failed. This phenomenon occurred because the categories involved in the training and testing sets were different. In this experiment, HALNet still exhibited better registration performance than other methods.

### 4.5. Ablation Experiment

#### 4.5.1. Hybrid Attention

To evaluate the impact of hybrid attention on registration performance, the attention module was removed as the first comparison method (M1) and the attention module in HALNet was replaced with a transformer [39] as the second comparison method (M2). For readability, HALNet was labeled as M0 in this experiment. The experiments still included the Unseen Shapes, Noise, and Unseen Categories. The results are shown in Table 4.

The results in the table indicated that the use of attention improved the registration performance of the network, and the hybrid attention proposed in this paper had a greater impact on registration performance improvement than the transformer. The higher network complexity of the transformer compared to hybrid attention may be one of the reasons for its weaker performance.

#### 4.5.2. Fully Connected Layer

This experiment compared the performance of using fully connected layers and SVD to solve rigid transformations. The experiment included the Unseen Shapes, Noise, and Unseen Categories. The fully connected layer was labeled as FCL. The results are shown in Table 5. The results indicated that using fully connected layers enabled registration network to achieve better registration performance.

## 5. Conclusions

For partial point cloud registration, HALNet is proposed. HALNet extracts local features of point clouds utilizing AGConv and CBAM and uses a hybrid attention mechanism to fuse the geometry of source and target point clouds. The hybrid attention mechanism is composed of self-attention and cross-attention, and its structure is simpler than the transformer which is also composed of self-attention and cross-attention. The ablation experiment proves that HALNet using hybrid attention has better registration performance than a transformer or HALNet used without an attention mechanism. The fully connected layer with learnable parameters is used to predict the rigid transformation instead of SVD. The ablation experiment shows that HALNet with a fully connected layer achieves better registration performance than SVD for the rigid transformation. HALNet was compared to five other registration methods with advanced registration performance, and HALNet showed excellent registration performance with robustness and generalization.

Since all the experiments in this paper were carried out on the ModeNet40 dataset, the performance on real scan data is unknown. In future research, HALNet will be applied to the dataset scanned in the real scene to test the viability of the method in the real scene. In addition, the simple structure of the hybrid attention mechanism leads to the discovery of detailed features to be improved, and future work can focus on designing better attention mechanisms.

## Figures and Tables

**Table 1:** Partial point cloud registration results in unseen shapes.

Model	RMSE(R)	MAE(R)	RMSE(t)	MAE(t)
DeepGMR	13.382577	8.870650	0.041859	0.029198
DCP-V2	5.478204	3.538027	0.026153	0.019312
IDAM	7.621095	3.713719	0.047063	0.024128
SCANet	3.465979	1.982889	0.014385	0.010212
VRNet	5.093359	3.364641	0.030701	0.022927
HALNet	3.096093	1.743890	0.022106	0.017327

**Table 2:** Partial point cloud registration results in noise.

Model	RMSE(R)	MAE(R)	RMSE(t)	MAE(t)
DeepGMR	13.352157	8.878562	0.041774	0.029208
DCP-V2	5.897242	3.899607	0.026251	0.019345
IDAM	7.472706	3.453048	0.043778	0.022701
SCANet	3.484932	2.003082	0.014277	0.010201
VRNet	5.478986	3.679850	0.030500	0.022766
HALNet	3.357249	1.948029	0.022781	0.017951

**Table 3:** Partial point cloud registration results in unseen categories.

Model	RMSE(R)	MAE(R)	RMSE(t)	MAE(t)
DeepGMR	14.122125	9.947880	0.043104	0.031322
DCP-V2	6.295135	4.116909	0.029018	0.021801
IDAM	8.044392	3.891389	0.048142	0.025395
SCANet	4.545772	2.933826	0.020703	0.014922
VRNet	5.947994	4.035112	0.033973	0.025189
HALNet	4.091592	2.616030	0.026665	0.020893

**Table 4:** The comparison experiment results for hybrid attention.

Condition	Method	RMSE(R)	MAE(R)	RMSE(t)	MAE(t)
Unseen Shapes	M1	3.934837	2.495351	0.025345	0.019687
	M2	3.469968	2.138049	0.023129	0.018045
	M0	3.096093	1.743890	0.022106	0.017327
Noise	M1	4.132365	2.701966	0.025643	0.019908
	M2	3.551702	2.234420	0.023199	0.018124
	M0	3.357249	1.948029	0.022781	0.017951
Unseen Categories	M1	5.165231	3.534752	0.030933	0.024414
	M2	4.227642	2.852501	0.028591	0.022475
	M0	4.091592	2.616030	0.026665	0.020893

**Table 5:** The comparison experiment results for fully connected layer.

Condition	Method	RMSE(R)	MAE(R)	RMSE(t)	MAE(t)
Unseen Shapes	SVD	4.905889	2.797221	0.0222372	0.016029
	FCL	3.096093	1.743890	0.022106	0.017327
Noise	SVD	5.034445	2.943128	0.022696	0.016189
	FCL	3.357249	1.948029	0.022781	0.017951
Unseen Categories	SVD	6.336225	4.035086	0.029551	0.022089
	FCL	4.091592	2.616030	0.026665	0.020893

## Data Availability

The data presented in this study are openly available in [32].
